# High-dose methotrexate in combination with interferons in the treatment of malignant pleural mesothelioma

**DOI:** 10.1038/sj.bjc.6690597

**Published:** 1999-08

**Authors:** M Halme, A Knuuttila, T Vehmas, L Tammilehto, M Mäntylä, J Salo, K Mattson

**Affiliations:** Department of Medicine, Division of Pulmonary Medicine and Clinical Physiology, and Departments of Radiology, Oncology and Thoracic and Cardiovascular Surgery, Helsinki University Central Hospital, Helsinki, HYKS, FIN-00029, Finland; Finnish Institute of Occupational Health, Helsinki, 00290, Finland

**Keywords:** high dose MTX, IFN-α, IFN-γ, pleural mesothelioma

## Abstract

Twenty six patients with pleural mesothelioma of UICC stage I–IV excluding M1 disease (46% of whom had stage I disease and 38% stage III disease) were treated intravenously with high dose MTX (3 g) and calcium folinate rescue three times at intervals of 2 weeks and three times at intervals of 3 weeks. Natural interferon (IFN)-α (3 MIU days 2–10) and recombinant IFN-γ 1b (50 μg m^−2^ on days 2, 6 and 10) were injected subcutaneously after each MTX dose. At the end of MTX treatment the IFNs were continued as maintenance therapy until disease progression. Seven partial responses were observed among 24 patients evaluable for response (response rate 29%, 95% confidence interval 13–51%). Median duration of response was 10 months (range 3–24 months). Median survival was 17 months and 1-year and 2-year survival rates 62% and 31% respectively. The toxicity of the chemo-immunotherapy was acceptable. Treatment was stopped in one patient who developed grade IV neurological toxicity. MTX dose reductions were rare (two patients with grade 1–2 renal toxicity). The combination of high dose MTX and IFN-α and IFN-γ is active against malignant pleural mesothelioma and well-tolerated. The survival rates are encouraging. © 1999 Cancer Research Campaign


*British Journal of Cancer* (1999) **80**(11), 1781–1785
© 1999 Cancer Research Campaign
Article no. bjoc.1999.0597

### High-dose methotrexate in combination with interferons in the treatment of malignant pleural mesothelioma

**M Halme[1], A Knuuttila[1], T Vehmas[2], L Tammilehto[5], M Mäntylä[3], J Salo[4] and K Mattson[1]**

[1]Department of Medicine, Division of Pulmonary Medicine and Clinical Physiology, and Departments of [2]Radiology, [3]Oncology and [4]Thoracic and Cardiovascular Surgery, Helsinki University Central Hospital, PO Box 340, FIN-00029 HYKS, Helsinki, Finland; [5]Finnish Institute of Occupational Health, 00290 Helsinki, Finland

**Summary** Twenty six patients with pleural mesothelioma of UICC stage I–IV excluding M1 disease (46% of whom had stage I disease and 38% stage III disease) were treated intravenously with high dose MTX (3 g) and calcium folinate rescue three times at intervals of 2 weeks and three times at intervals of 3 weeks. Natural interferon (IFN)-α (3 MIU days 2–10) and recombinant IFN-γ 1b (50 µg $m^{-2}$ on days 2, 6 and 10) were injected subcutaneously after each MTX dose. At the end of MTX treatment the IFNs were continued as maintenance therapy until disease progression. Seven partial responses were observed among 24 patients evaluable for response (response rate 29%, 95% confidence interval 13–51%). Median duration of response was 10 months (range 3–24 months). Median survival was 17 months and 1-year and 2-year survival rates 62% and 31% respectively. The toxicity of the chemo-immunotherapy was acceptable. Treatment was stopped in one patient who developed grade IV neurological toxicity. MTX dose reductions were rare (two patients with grade 1–2 renal toxicity). The combination of high dose MTX and IFN-α and IFN-γ is active against malignant pleural mesothelioma and well-tolerated. The survival rates are encouraging.

**Keywords:** high dose MTX; IFN-α; IFN-γ; pleural mesothelioma

Malignant mesothelioma is an aggressive tumour arising from serous surfaces. It is usually related to asbestos exposure and generally resistant to conventional chemotherapy.

Only a few single chemotherapeutic agents have been reported to produce response rates greater than 20% in patients with pleural mesothelioma. Among the most promising agents have been anthracyclines (detorubicin and pirarubicin), ifosfamide, mitomycin and some antifolates such as methotrexate (MTX) in high doses and edatrexate (Ong and Vogelzang, 1996; Ryan et al, 1998).

High-dose MTX was first reported to induce objective responses in patients with mesothelioma by Dimitrov et al (1982) who treated nine patients with MTX at 1500 mg $m^{-2}$ as a continuous infusion with citrovorum rescue and vincristine. In this early study tumour response was assessed without computerized tomography using chest radiographs and sonograms. Three complete responses (CR) and three partial responses (PR) were achieved, while three patients showed no change (NC), but had less need of fluid removal. Two of the responders (PR) had not received any prior therapy, while the others had received two chemotherapeutic agents or one chemotherapy agent plus radiotherapy and surgical debulking before starting high-dose MTX. Duration of response and survival of the responders were 2–23 months and 5–35 months respectively (Dimitrov et al, 1982). Solheim et al (1992) reported on 63 patients with pleural mesothelioma who were treated with 4–8 courses of MTX at 3 g with citrovorum rescue. Of 60 patients evaluable for response (assessed using computerized tomography), 37% achieved PR ($n$ = 21) or CR ($n$ = 1). Median survival for all patients was 11 months and 40% of the patients were alive after 1 year. Toxicity was acceptable, with one toxic death and five patients (8%) discontinuing treatment due to toxicity (Solheim et al, 1992).

In experimental studies both natural and recombinant interferon (IFN)-α have been reported to have inhibitory effects on human mesothelioma xenograft lines (Sklarin et al, 1988; Ohnuma et al, 1993). In a clinical study of 13 patients with pleural mesothelioma one response was achieved using systemic administration of recombinant IFN-α2b (Ardizzoni et al, 1994), and in another study of 25 mesothelioma patients recombinant IFN-α2a induced one CR and two PRs (response rate 12%) (Christmas et al, 1993). However, no responses were noted when 14 mesothelioma patients were administered recombinant IFN-β systemically (Von Hoff et al, 1990), although eight CRs and nine PRs (objective response rate 20%) were achieved using intrapleurally instilled recombinant IFN-γ to treat 89 patients with mesothelioma. Most of these responders had early stage disease (Boutin et al, 1994). After the Boutin report, Zeng et al (1993) demonstrated a large range of responses to recombinant human IFN-γ in experimental studies on 32 human mesothelioma cell lines.

The antiproliferative effects of IFN-α and IFN-γ both alone and in combination with various chemotherapeutic agents on mesothelioma cell lines has been demonstrated in the earlier studies of our group (Hand et al, 1991). After the Solheim report on high-dose MTX therapy for patients with pleural mesothelioma, we tested the combination of IFN-α and IFN-γ with MTX in four human mesothelioma cell lines. IFN-α and IFN-γ in combination augmented the response of the cell lines to MTX better than either

*Received 22 July 1998*
*Accepted 20 February 1999*

*Correspondence to:* M Halme

IFN alone. Natural IFN-β was also compared to IFN-α and IFN-γ and it was found to have a similar sensitivity profile to that of IFN-α (Hand et al, 1995).

The aim of this clinical phase II study was to investigate the activity and to evaluate the toxicity of the combination of high-dose MTX with IFN-α and IFN-γ in patients with malignant pleural mesothelioma.

### MATERIALS AND METHODS

### Patient selection

Patients with previously untreated, histologically confirmed (by a panel of pathologists) malignant pleural mesothelioma were eligible for the study. Additional entry criteria were age 18–70 years, performance status WHO 0–1, tumour classification T1–3N0–3M0 as verified by computerized tomography (CT) within 14 days, measurable tumour lesion (assessed using CT), adequate bone marrow reserve (WBC > $3 \times 10^9$ $l^{-1}$ and platelet count > $100 \times 10^9$ $l^{-1}$, adequate liver and renal function (serum creatinine and transaminase levels < twice the upper normal limit). Patients with other malignant disease (except stage I cervix carcinoma), severe cardiac disease, severe mental disturbance or a known seizure disorder were excluded. All patients gave informed written consent before starting the treatment. The study was approved by the Ethics Committee of the Division of Pulmonary Medicine and Clinical Physiology, Department of Medicine at the Helsinki University Central Hospital.

### Treatment schedule

Patients were assigned to receive six doses of MTX with calcium folinate rescue in combination with subcutaneous injections of IFN-α and IFN-γ. Chest radiographs and laboratory tests including total blood cell count, transaminases, alkaline phosphatase, serum bilirubin, potassium, sodium, C-reactive protein and urine analysis were checked before each MTX infusion and at the end of the treatment. Response was evaluated from CT scans of the chest and upper abdomen after three doses and six doses of MTX and at any suspicion of tumour progression. The treatment was discontinued if progressive disease (PD) was detected. For patients who achieved objective responses the treatment could be continued beyond six doses. Toxicity was evaluated using WHO criteria. All patients were re-evaluated 1, 3 and 6 months after discontinuing treatment and thereafter when progression was suspected using above-mentioned laboratory tests and CT. Survival was calculated from the first MTX dose.

#### *Methotrexate*

Urine had to be kept alkaline (pH > 8.0) to secure adequate excretion of soluble MTX. The day before MTX treatment patients received 200 ml bicarbonate solution (500 mmol $l^{-1}$) as well as started taking bicarbonate tablets (3 g) four times a day for 5 days. The pH of urine was measured four times per 24 h and the bicarbonate dose was increased if the urinal pH dropped below 8.0.

Methotrexate (Trexan^R^) 3 g was administered on days 0, 14, 28, 49, 70 and 91. Sufficient hydration was maintained with a total fluid intake of at least 2500 ml every 24 h until the MTX concentration fell below 0.1 μmol $l^{-1}$. Calcium folinate (Antrex^R^) rescue was initiated 24 h after the start of MTX treatment using 15 mg (either orally or intravenously) every 6 h for a minimum of 11 doses. Further treatment was administered if the MTX concentration in serum had not dropped below 0.1 μmol $l^{-1}$ 96 h after the MTX infusion. Calcium folinate treatment was then continued until the MTX level was below 0.08 μmol $l^{-1}$.

#### *Interferons*

Interferon treatment was only started after the MTX concentration in serum had decreased to 0.2 mM. Natural IFN-α (Wellferon^R^) 3 MIU was administered subcutaneously on days 2–10 after each dose of MTX. It was continued at the same dose three times a week after the MTX treatment had finished until disease progression.

Recombinant IFN-γ 1b (Imukin^R^) 50 μg $m^{-2}$ (maximum 100 μg) was administered subcutaneously on days 2, 6 and 10 after each dose of MTX. When the MTX treatment was completed IFN-γ was continued at the initial dose once a week until disease progression.

### Response criteria

Tumour response was assessed from CT scans of the chest and upper abdomen according to the World Health Organization (WHO) criteria. All scans were reviewed by a radiologist.

Response was assessed according to the following criteria. CR was defined as the disappearance of all tumour tissue and pleural exudate. PR required the disappearance of at least 50% of tumour tissue within all marker lesions. Progressive disease required an increase of at least 25% in tumour size. NC indicated stable disease with less change in the tumour size than PR or PD.

### Statistical analysis

Differences in patient characteristics between responders and non-responders were analysed using Fisher's exact test. Confidence intervals for the response rates were computed using a binomial distribution. Survival was estimated using the life-table method.

### RESULTS

Twenty six patients met the entry criteria between March 1992 and May 1997. Clinical characteristics of the patients are given in Table 1. According to the staging system proposed by UICC (Union Internationale Contre le Cancer) (UICC 1992) 46% (12/26) of the patients had stage I disease. Another 46% (12/26) of the patients had more advanced stage III–IV disease due to several T3 tumours and two patients with grade 3 nodal status. Subtype analysis revealed epithelial histology in 65% (17/26) of the patients. One patient had a sarcomatoid tumour and the rest had mixed type tumours. Twenty-one patients (81%) were known to have been exposed occupationally to asbestos.

Between one and six doses of MTX (mean 5.1) were given to each patient. Eighteen patients received all six cycles of scheduled treatment. Two of these patients received additional six doses of MTX because of continuing response. Two patients received only one dose of MTX. Both patients had rapidly progressive disease and at the time of the second dose the other one had respiratory infection as well. Their treatment was decided to be discontinued before the second dose due to poor performance status. These patients were determined non-evaluable for response analysis. Reduced doses (1.5–2 g) were given to two patients.

*© 1999 Cancer Research Campaign*

**Table 1** Patient characteristics

| | |
|---|---|
| *N* | 26 |
| Sex | |
| Males | 23 |
| Females | 3 |
| Age (years) | |
| Mean | 55 |
| Range | 43–70 |
| Histology | |
| Epithelial | 17 |
| Sarcomatoid | 1 |
| Mixed | 8 |
| Clinical stage | |
| I | 12 |
| II | 2 |
| III | 10 |
| IV | 2 |
| Performance status (WHO Grade) | |
| 0 | 10 |
| 1 | 16 |

**Table 2** Toxic effects during methotrexate + IFN treatment

| | Grade (WHO criteria) | | | |
|---|---|---|---|---|
| **Toxicity** | **1** | **2** | **3** | **4** |
| Haematological | | | | |
| Anaemia | 2 | 3 | 0 | 0 |
| Leukopenia | 7 | 12 | 7 | 0 |
| Thrombocytopenia | 1 | 0 | 0 | 0 |
| Gastrointestinal | 7 | 2 | 3 | 0 |
| Renal | 1 | 2 | 0 | 0 |
| Neurological | 0 | 0 | 0 | 1 |
| Cutaneous | 0 | 2 | 0 | 0 |

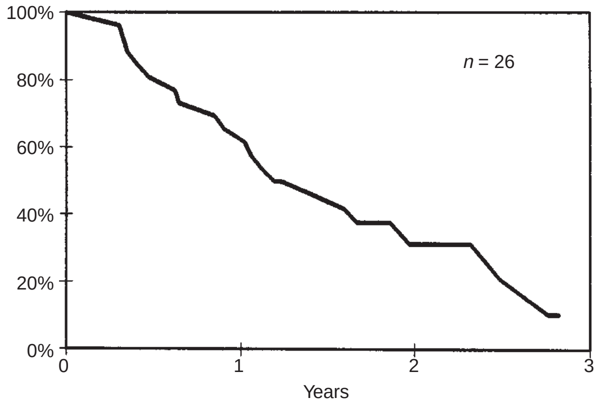

**Figure 1** Survival of all patients

Twenty-three patients were given additional therapies for tumour progression within 1–22 months (mean 5.9) of discontinuing MTX. All these patients received radiotherapy (palliative doses of 20–30 Gy for 14 patients and 50 Gy for nine patients). Seven patients received other chemotherapy (docetaxel five patients, camptothecin one patient, paclitaxel + carboplatin one patient). Two of the patients who were not given any additional therapy died early of progressive disease.

### Response to treatment

Twenty four (92.3%) patients were evaluable for tumour response. None of the patients achieved CR but there were seven PRs (objective response rate 29%, [95% confidence interval (CI) 13–51%]). Four patients in the responder group had epithelial tumours while the others had tumours of mixed histology. There were 13 epithelial, three mixed and one sarcomatoid tumour in the non-responder group. Five of the responders had stage I disease and two had stage III disease. In the non-responder group seven patients had stage I disease, one patient stage II disease, eight patients stage III disease and one patient stage IV disease. Three of the responders had a performance status of WHO grade 0 and four of grade 1, as opposed to seven and 10, respectively, in the non-responder group. All except one of the patients evaluable for tumour response received radiotherapy after MTX treatment. Other chemotherapy was given to three responders and four non-responders. There were no statistical differences in tumour histology, tumour stage, performance status or additional therapies between responders and non-responders. The maximum response was observed after three cycles in five patients, although two patients only responded after six cycles. Median duration of response was 10 months ranging from 3 to 24 months. Fourteen patients showed NC, and three patients PD after three cycles. Nine of the NC patients retained the same status after six cycles. Median time to progression for all patients was 4 months (range 2 weeks to 24 months).

### Survival

All 26 patients meeting the entry criteria were included in the survival analysis. Median survival of all patients was 17 months (range 3.5–36+ months) and 1- and 2-year survival rates were 62% and 31% respectively (Figure 1). One-year survival rates for the responders ($n = 7$) and non-responders ($n = 17$) were 85.7% and 64.7% respectively (not significant), and two-year survival rates 57.1% and 11.8% respectively ($P < 0.05$). Five patients (two in the responder group) were alive at the time of evaluation.

### Toxicity

All patients were evaluable for toxicity (Table 2). Leukopenia was the most common haematological toxicity occurring in all patients. Seven patients (27%) had WHO grade 3 leukopenia, while the remaining 19 had grade 1–2. WHO grade 1–2 anaemia occurred in five patients (19%). WHO grade 1 thrombocytopenia was only detected in one patient.

Fourteen patients suffered from non-haematological toxicity during the chemo-immunotherapy. WHO grade 3 gastrointestinal toxicity (elevation in transaminase levels) occurred in three (12%) patients while grade 1–2 toxicity occurred in nine patients. Two patients had renal toxicity (elevation in creatinine levels) of WHO grade 2 and one patient of grade 1. Renal toxicity lead to reductions in the MTX dose for two patients (1.5 g and 2 g).

Two patients suffered from WHO grade 2 rash. One patient developed hemiparesis 10 days after the third MTX dose. This adverse event was graded as WHO grade 4 neurological toxicity and led to the discontinuation of treatment despite a PR. A CT of the brain, electroencephalography and a thorough neurological evaluation did not provide any explanation of this event which was

© 1999 Cancer Research Campaign reversible. Later the patient received palliative radiotherapy after PD and died 3 months later of progressive disease. MTX-related mucositis was not observed in any of our patients.

During IFN maintenance therapy toxicity was mild and transient. WHO grade 1 leukopenia was detected in three patients. Elevation of transaminase levels was detected in four patients (one grade 3, two grade 2 and one grade 1). Flu-like symptoms were common side-effects of IFN treatment but required no dose adjustments. They were usually clinically manageable, although one patient stopped administering IFN because of this side-effect.

### DISCUSSION

This study is the first clinical report on the combination of MTX and IFN used against mesothelioma. The objective response rate 29% (95% CI 13–51%), confirms MTX as an active agent, but in the light of Solheim's study which achieved a response rate of 37% using the same MTX dose and time schedule, no benefit can be attributed to the addition of IFN in our regime (Solheim et al, 1992). Our slightly lower response rate cannot be attributed to dosage either because we gave full doses of MTX to all except two patients (who had elevations in creatinine levels as a result of the first dose of MTX). One reason could be that there was more advanced disease in our patient population than in Solheim's population. Forty-six per cent of our patients had stage III–IV disease compared to 10% in the Solheim study (Solheim et al, 1992). However, the response rate achieved by Solheim lies within the 95% confidence interval of our response rate.

The survival rates in our study are more favourable than in the Solheim study. When analysing the survival figures we must, however, take into account the other treatments given to our patients. Seven of our 26 patients received other chemotherapy after finishing the MTX regime which may have affected survival. Radiotherapy was given to 23 patients with bulky disease, mainly for pain alleviation. Importantly, however, the additional treatments given to our patients were comparable for responder and non-responder groups and cannot explain the significantly longer survival time in the responder group. Solheim did not record survival for responders and non-responders, but the median survival of all his patients was shorter than in our study (11 vs 17 months) despite of the more advanced disease in our patient population.

MTX at conventional doses has been combined successfully with other chemotherapeutic agents in the treatment of mesothelioma. Hunt et al (1996) reported two CRs among nine responders (response rate 53%, 95% CI 28–77%) in a study of 17 mesothelioma patients treated with cisplatin and vinblastine combined with MTX at a dose of 30 mg $m^{-2}$, which exceeds the response achieved using MTX alone. Doxorubicin is another cytotoxic agent which has been reported to induce objective responses of at least 30% of mesothelioma patients, when used in combination. The combination of cisplatin, cyclophosphamide and doxorubicin has been reported to produce a response rate of 30% (Shin et al, 1995). Two CRs and one PR were achieved using high dose doxorubicin (90 mg $m^{-2}$) combined with cisplatin for patients with mesothelioma in a study reported by Stewart et al. Although this was a pilot study with only four patients, the prolonged survival of these patients (> 4 years in one patient) is a promising result (Stewart et al, 1994). Doxorubicin at a dose of 75 mg $m^{-2}$ combined with ifosfamide produced seven PRs (response rate 32%, 95% CI 13–51%) in 22 patients, although median duration of response as well as median survival were short, 6 months and 7 months respectively (Dirix et al, 1994).

Recombinant IFN-α2a has been combined with doxorubicin in a clinical trial for 25 mesothelioma patients. Four PRs were achieved, giving a response rate of 16% (Upham et al, 1993). Augmentation of the efficacy of the chemotherapeutic agent by IFN was not therefore confirmed by this study. Tansan et al (1994) combined recombinant IFN-α2b with cisplatin and mitomycin C and achieved only two PRs among 19 evaluable mesothelioma patients. They also concluded that the addition of IFN-α to cytotoxic agents did not result in an objective response higher than previously reported for the agents alone (Tansan et al, 1994). Furthermore, Pass et al (1995) reported a response rate of 19% in 36 mesothelioma patients after cisplatin, oral tamoxifen and subcutaneously administrated IFN-α2b given four times a week. However, weekly cisplatin combined with subcutaneously administered IFN-α2a produced ten PRs among 25 mesothelioma patients (response rate 40%, 95% CI 20–60%), indicating that IFN can have an additive or synergistic effect on cisplatin. Median survival of the responders was significantly higher than in the non-responder group (25 vs 8 months) (Soulie et al, 1996).

Response evaluation in mesothelioma is not standardized and only recently has a staging classification been recommended that allows inter-trial comparisons (Rusch, 1995). The relatively small studies so far reported have therefore been difficult to compare and no data could have been regarded as baseline for future trials. The two MTX studies are exceptions in this context. Both Solheim's report on 63 patients and our report on 26 patients indicate that MTX at a relatively high dose is active against mesothelioma. The survival figures in our study are encouraging. The contributory effect of IFN on survival benefit remains open; a proper assessment would require a randomized trial.

### REFERENCES

Ardizzoni A, Pennucci MC, Castagneto B, Mariani GL, Cinquegrana A, Magri D, Verna A, Salvati F and Rosso R (1994) Recombinant interferon alpha-2b in the treatment of diffuse malignant pleural mesothelioma. *Am J Clin Oncol (CCT)* **17**: 80–82

Boutin C, Nussbaum E, Monnet I, Bignon J, Vanderschueren R, Guerin J-C, Menard O, Mignot P, Dabouis and Douillard J-Y (1994) Intrapleural treatment with recombinant gamma-interferon in early stage malignant pleural mesothelioma. *Cancer* **74**: 2460–2467

Christmas TI, Manning LS, Garlepp MJ, Musk AW and Robinson BW (1993) Effect of interferon-alpha 2a on malignant mesothelioma. *J Interferon Res* **13**: 9–12

Dimitrov NV, Egner J, Balcueva E and Suhrland LG (1982) High-dose methotrexate with citrovorum factor and vincristine in the treatment of malignant mesothelioma. *Cancer* **50**: 1245–1247

Dirix LY, van Meerbeeck J, Schrijvers D, Corthouts B, Prove A, van Marck E, Vermeire P and van Oosterom AT (1994) A phase II trial of dose-escalated doxorubicin and ifosfamide/mesna in patients with malignant mesothelioma. *Ann Oncol* **5**: 653–655

Hand AM, Husgafvel-Pursiainen K, Tammilehto L, Mattson K and Linnainmaa K (1991) Malignant mesothelioma: the antiproliferative effect of cytokine combinations on three human mesotelioma cell lines. *Cancer Lett* **58**: 205–210

Hand AM, Pelin K, Mattson K and Linnainmaa K (1995) Interferon (IFN)-α and IFN-γ in combination with methotrexate: in vitro sensitivity studies in four human mesothelioma cell lines. *Anti-Cancer Drugs* **6**: 77–82

Hunt KJ, Longton G, Williams MA and Livingston RB (1996) Treatment of malignant mesothelioma with methotrexate and vinblastine, with or without platinum chemotherapy. *Chest* **109**: 1239–1242

Ohnuma T, Szrajer L, Holland JF, Kurimoto M and Minowada J (1993) Effects of natural interferon alpha, natural tumor necrosis factor alpha and their combination on human mesothelioma xenografts in nude mice. *Cancer Immunol Immunother* **36**: 31–36

 *© 1999 Cancer Research Campaign*

Ong S and Vogelzang NJ (1996) Chemotherapy in malignant pleural mesothelioma: a review. *J Clin Oncol* **14**: 1007–1017

Pass HW, Temeck BK, Kranda K, Steinberg SM and Pass HI (1995) A phase II trial investigating primary immunochemotherapy for malignant pleural mesothelioma and the feasibility of adjuvant immunochemotherapy after maximal cytoreduction. *Ann Surg Oncol* **2**: 214–220

Rusch V (1995) A proposed new international TNM staging system for malignant pleural mesothelioma. *Chest* **108**: 1122–1128

Ryan CW, Herndon J and Vogelzang NJ (1998) A review of chemotherapy trials for malignant mesothelioma. *Chest* **113**: 66S–73S

Shin DM, Fossella FV, Umsawasdi T, Murphy WK, Chasen MH, Walsh G, Komaki R, McMurtrey MJ and Hong WK (1995) Prospective study of combination chemotherapy with cyclophosphamide, doxorubicin, and cisplatin for unresectable or metastatic malignant pleural mesothelioma. *Cancer* **76**: 2230–2236

Sklarin NT, Chaninian AP, Feuer EJ, Lahman LA, Szrajer L and Holland JF (1988) Augmentation of activity of cis-diamminedichloroplatinum(II) and mitomycin C by interferon in human malignant mesothelioma xenografts in nude mice. *Cancer Res* **48**: 64–67

Solheim OP, Säter G, Finnanger AM and Stenwig AE (1992) High-dose methotrexate in the treatment of malignant mesothelioma of the pleura. A phase II study. *Br J Cancer* **65**: 956–960

Soulie P, Ruffie P, Trandafir L, Monnet I, Tardivon A, Terrier P, Cvitkovic E, Le Chevalier T and Armand JP (1996) Combined systemic chemoimmunotherapy in advanced diffuse malignant mesothelioma: report of phase I–II study of weekly cisplatin/interferon α-2a. *J Clin Oncol* **14**: 878–885

Stewart DJ, Gertler SZ, Tomiak A, Shamji F, Goel R, Evans WK (1994) High dose doxorubicin plus cisplatin in the treatment of unresectable mesotheliomas: report of four cases. *Lung Cancer* **11**: 251–258

Tansan S, Emri S, Selcuk T, Koc Y, Hesketh P, Heeren T, McCaffrey RP and Baris YI (1994) Treatment of malignant pleural mesothelioma with cisplatin, mitomycin C and alpha interferon. *Oncology* **51**: 348–3451

UICC (1992) *International Union against Cancer: TNM Classification of Malignant Tumours*. Springer Verlag: Berlin.

Upham JW, Musk AW, van Hazel G, Byrne M and Robinson BW (1993) Interferon alpha and doxorubicin in malignant mesothelioma: a phase II study. *Aust NZ J Med* **23**: 683–687

Von Hoff DD, Metch B, Lucas JG, Balcerzak SP, Grunberg SM and Rivkin SE (1990) Phase II evaluation of recombinant interferon-beta (IFN-β ser) in patients with diffuse mesothelioma: a Southwest Oncology Group study. *J Interferon Res* **10**: 531–534

Zeng L, Buard A, Monnet I, Boutin C, Fleury J, Saint-Etienne L, Brochard P, Bignon J and Jaurand MC (1993) In vitro effects of recombinant human interferon gamma on human mesothelioma cell lines. *Int J Cancer* **55**: 515–520

*© 1999 Cancer Research Campaign*
